# Redefining pain management: investigating the efficacy and safety of erector spinae plane block and oblique subcostal transversus abdominis plane block in laparoscopic cholecystectomy – a meta analysis of randomized controlled trials

**DOI:** 10.1186/s12871-025-03059-1

**Published:** 2025-04-16

**Authors:** Syed Zia Saleem, Syed Muhammad Muneeb Akhtar, Areeba Fareed, Afsana Ansari Shaik, Muhammad Sohaib Asghar

**Affiliations:** 1 Department of Medicine, Dow University of Health Sciences, Karachi, Pakistan; 2 Department of Medicine, Karachi Medical and Dental College, Karachi, Pakistan; 3 Mayo Clinic, Rochester, MN USA; 4 AdventHealth Sebring, Sebring, FL USA

**Keywords:** Erector spinae plane block, Oblique subcostal transversus abdominis plane block, Postoperative pain, Laparoscopic cholecystectomy, Meta-analysis

## Abstract

**Background:**

Pain following laparoscopic cholecystectomy plays a pivotal role in determining the quality of patient recovery. Considering the opioid crisis, exploration of alternative approaches, such as regional blocks, including erector spinae plane block (ESPB) and oblique subcostal transversus abdominis plane block (OSTAPB), has garnered considerable attention due to their promising outcomes in clinical trials.

**Objective:**

Our aim is to provide a robust analysis which reflects the most current evidence for the effectiveness and safety of ESPB by comparing it to OSTAPB in adult patients undergoing laparoscopic cholecystectomy.

**Methods:**

An extensive search was performed in the PubMed, Medline, and Cochrane Library databases from inception to June 1st 2023. Mean difference (SMD), and 95% confidence intervals (CIs) were calculated for continuous outcomes, Risk ratios (RR) were calculated for dichotomous outcomes. All statistical analyses were performed using R Statistical Software and meta package v4.17–0.

**Results:**

A total of 5 RCTs including 372 participants were included in this meta-analysis. Pooled analysis of overall postoperative pain scores at 12 and 24 h showed ESPB to be superior to OSTAPB [MD = -0.67; 95% CI: (-0.95 to -0.39); *p* < 0.001, I^2^ = 72%]. ESPB also showed significantly lesser opioid consumption at 24 h postoperatively [MD = -5.36; 95% CI: (-8.56 to –2.15); *p* < 0.001, I^2^ = 96%], while intraoperative opioid consumption {MD = -0.46; 95% CI: (-1.27 to –0.36); *p* = 0.27, I^2^ = 0%} and postoperative nausea and vomiting were not significantly different between the two groups {RR = 0.40, 95% CI (0.10 to 1.56), *p* = 0.19; I^2^ = 56%}.

**Conclusion:**

In summary, the erector spinae plane block (ESPB) appears to be the preferred option for acute postoperative pain and opioid reduction in adults undergoing laparoscopic cholecystectomy.

**Supplementary Information:**

The online version contains supplementary material available at 10.1186/s12871-025-03059-1.

## Introduction

Laparoscopic cholecystectomy (LC) is a minimally invasive surgical procedure for removing the gallbladder. Its advantages of shorter recovery time, less postoperative pain, and a lower risk of complications have made it more popular than open surgery [1]. However, despite having significantly lesser postoperative pain, it still occurs to some degree in LC, which includes somatic pain from the incision sites, local visceral pain, parietal pain, and referred visceral pain [2–4].

Anesthesiologists play a pivotal role in ensuring optimal relief of post-operative pain, and peripheral nerve blocks serve as a valuable tool to achieve this objective. The utilization of ultrasound technology has conferred added benefits to this technique, facilitating a more streamlined and efficient approach. Comparative analyses have demonstrated the superiority of nerve blocks over conventional pain management modalities such as nonsteroidal anti-inflammatory drugs (NSAIDs) and opioids, given their targeted blockade of pain pathways at specific sites. [5], pain tolerance varies substantially among individuals, and those with a lower threshold often encounter difficulties managing their pain, even when utilizing opioids and nonsteroidal anti-inflammatory drugs (NSAIDs) at maximal doses. Additionally, the application of these agents may lead to delayed recovery and trigger gastritis, ileus, nausea, and vomiting, all of which can exacerbate patients'overall condition [3].

Conversely, ultrasound-guided plane blocks, such as transverse abdominis plane block (TAPB), oblique subcostal transverse abdominis plane block (OSTAPB), and erector spinae plane block (ESPB), have demonstrated the capability of reducing the requirement for opioids and NSAIDs throughout both the intra-operative and post-operative phases [6, 7].

Among these techniques, the use of ESPB and OSTAPB as part of multimodal analgesia is increasing progressively in various surgeries, one of which is in laparoscopic cholecystectomy due to their effectiveness in mitigating post-operative pain as evidenced by multiple published studies [8, 9].

ESPB blocks are a relatively new technique and in particular have shown a remarkable analgesic effect, with the block extending to cover several dermatomes, thereby producing a broader coverage area. It precisely targets not only the ventral rami and dorsal rami of the spinal nerves, but also the rami communicantes. Upon injecting the anesthetic agent, the results have shown notable cranial and caudal extension over multiple levels of dermatomes, making it a promising approach [10, 11]. While, OSTAPB blocks have been shown to target the sensory innervation of the abdominal wall, thereby reducing pain scores in patients undergoing laparoscopic cholecystectomy [12]. conclusion, by targeting specific nerves, ultrasound-guided techniques like OSTAPB, and ESPB blocks can significantly decrease postoperative pain and reduce the need for opioids and NSAIDs, which have several adverse effects that can hinder patient recovery.

Recently, in a meta-analysis by Yang et al. [13] compared ESPB with a control group and other blocks for adults undergoing LC, however for the comparison between ESPB and OSTAPB only a limited number of studies were included, while only doing meta-analysis of postoperative opioid consumption at 24 h outcome. This highlights the need for more comprehensive evaluation of these two techniques to form a consensus.

The primary goal of this meta-analysis is to provide an up-to-date and comprehensive synthesis of the latest evidence available regarding the comparative efficacy and safety of Erector Spinae Plane Block (ESPB) and Oblique Subcostal Transversus Abdominis Plane Block (OSTAPB) in adult patients who are undergoing laparoscopic cholecystectomy. It addresses following key questions:

1. What is the comparative efficacy of Erector Spinae Plane Block (ESPB) and Oblique Subcostal Transversus Abdominis Plane Block (OSTAPB) in terms of postoperative pain scores at 12 and 24 h following laparoscopic cholecystectomy?
2. How does postoperative opioid consumption at the 24-h mark differ between patients who receive Erector Spinae Plane Block (ESPB) and those who receive Oblique Subcostal Transversus Abdominis Plane Block (OSTAPB) during laparoscopic cholecystectomy?
3. What are the differences in intraoperative opioid consumption between patients undergoing laparoscopic cholecystectomy with Erector Spinae Plane Block (ESPB) versus Oblique Subcostal Transversus Abdominis Plane Block (OSTAPB)?
4. Does the incidence of postoperative nausea and vomiting (PONV) vary between individuals who undergo laparoscopic cholecystectomy with Erector Spinae Plane Block (ESPB) compared to those who receive Oblique Subcostal Transversus Abdominis Plane Block (OSTAPB)?

## Methods

### Data sources and search

A comprehensive literature search was conducted in the PubMed, Medline and Cochrane Library databases before May 2023 to identify relevant studies. Detailed search strategy is provided in Supplementary Table S1.

We applied no language restrictions and included studies in non-English language, and the relevant data were translated for interpretation using Google translate service. The relevant literature's references were carefully checked for potential eligible studies by the help of automated tools like End Note. Disagreements were resolved through consensus.

### Inclusion and exclusion criteria

The inclusion criteria for eligibility were as follows: (a) double-arm studies, (b) prospective randomized control trial (RCTs) comparing ESPB with OSTAPB block, (c) adult population going under laparoscopic cholecystectomy (LC) (d) outcomes of interest post operative pain scores, postoperative opioid consumption at 24 h, intraoperative opioid consumption, postoperative nausea (PON) and vomiting (POV) were reported (see Supplementary Table S2). The exclusion criteria included: (a) studies comparing ESPB with no block or other regional blocks in adults, (b) pediatric population, surgeries other than LC, (c) non- randomized trials, review articles, case reports, case series, editorials, abstracts, reviews, comments and letters, expert opinions, studies without original data and duplicate publications; (d) and studies that lacked a OSTAPB group for comparison.

### Data extraction

Two investigators (SMMA and SZS) independently extracted the following information from each included study: study characteristics (first author, year of publication, country, sample size and study type) surgical procedure, surgery duration, ESPB block level, participant baseline characteristics, post operative pain scores, postoperative opioid consumption at 24 h, intraoperative opioid consumption, postoperative nausea (PON) and vomiting (POV). Any discrepancy between data extractions was resolved by discussion with the third author (AAS).

### Quality assessment

The included RCTs were evaluated for quality using revised Cochrane Risk of Bias assessment tool (ROB2) [14]. Six components were assessed: (a) random sequence generation, (b) allocation concealment, (c) blinding of participants and personnel, (d) blinding of outcome assessment, (e) incomplete outcome data, and (f) selective reporting. According to whether the included studies fully meet the above criteria, we assessed the quality of trials. All items were independently assessed by two investigators (SMMA and SZS), with consensus reached after deliberation.

### Statistical analysis

The meta-analysis was carried out based on the guidelines of the preferred reporting items for systematic reviews and meta-analyses (PRISMA) statements [15]. All statistical analyses were performed using R Statistical Software [16] and meta package v4.17–0 [17]. For dichotomous outcomes we calculated Risk ratios (RR). To assess continuous variables, we calculated the mean difference (MD) and its respective 95% confidence interval and used inverse variance method. In cases where the continuous variables were presented as a median with a range (minimum to maximum) or an interquartile range, we used Luo and Wan's formula [18] to estimate the mean and standard deviation. The meta-analytical method used for the continuous outcome is the inverse variance method, with a restricted maximum-likelihood estimator for tau^2 as suggested by Veroniki et al. 2016 [19]. For binary outcomes, the Mantel–Haenszel method is utilized and incorporates the Paule-Mandel estimator for tau^2 as recommended by Veroniki et al. 2016. [19] If I^2^ is 0% then we used fixed effect model otherwise random effect model was used. The included studies measured the pain scores at rest or during coughing or active movement using either the visual analogue scale (VAS) or numeric rating scale (NRS), which were standardized to a 0–10 scale. Both the VAS and NRS were regarded as equivalent. Studies reporting outcomes within a time range we took the upper limit of the time reported for analysis.

To assess potential statistical heterogeneity among trials, the Higgins I^2^ statistics were used. The I^2^ statistic reveals the percentage of variation between studies that is due to heterogeneity rather than chance or sampling error. An outcome of over 75% indicates considerable heterogeneity. When the heterogeneity was high, subgroup analysis or sensitivity analysis was used to identify sources of heterogeneity. The results of meta-analysis were visually examined by forest plot. The *p* < 0.05 was considered statistically significant.

## Results

### Study characteristics

**Fig. 1:**
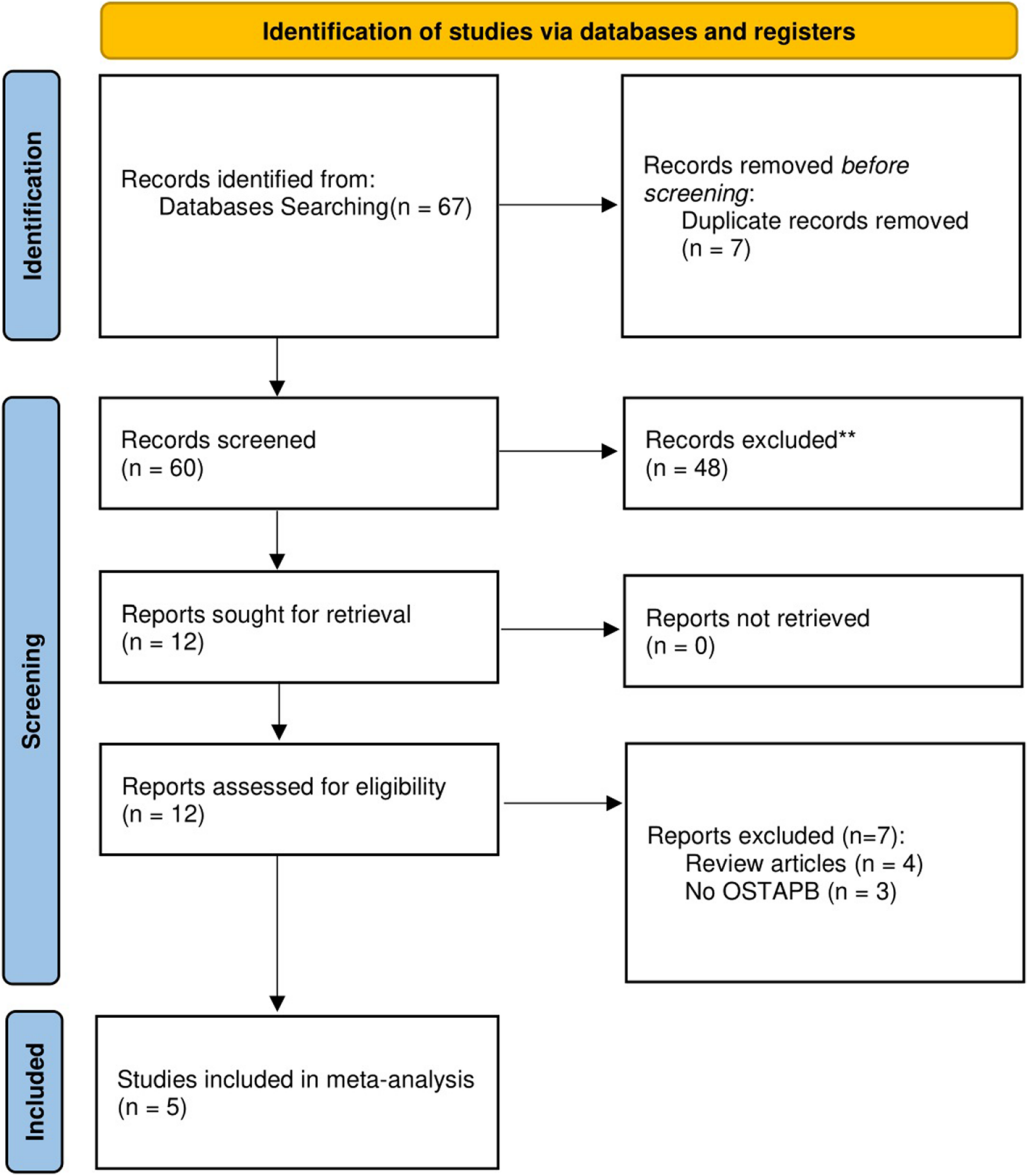
PRISMA Flow Diagram of the Literature Search Process

The study selection process is illustrated in Fig. 1, which presents a thorough flowchart. Following the preliminary literature search, a total of 67 articles were identified. After removing duplicates, the articles underwent a shortlisting process based on title, followed by abstract and full-text review. In total, 5 studies [20–24] deemed appropriate for inclusion in this meta-analysis.

The main characteristics of the included trials are presented in Table 1. The mean age ranged from 36.67 to 51.1 years in the ESPB group and from 37.5 to 50 years in the OSTAPB group, the reported mean surgery time across all the studies ranged from 42.6 to 120 min (about 2 h) in the ESPB group and 41.3 to 100 min (about 1 and a half hours) in the OSTAPB group.

**Table 1:** Study characteristics of the included studies

First Author and Study Year	Study location	Study design	Groups	No. Of Participants (n)	Mean age, (years)	Gender (male/female)	ASA Status I/II	BMI (kg/m2)	Duration of surgery (min)	ESPB Block Level
Altiparmak et al. [20] 2019	Muğla, Turkey	Prospective, Single-blinded, Randomized, Controlled study	ESPB OSTAPB	34 34	51.1 ± 12.3 53.1 ± 14.7	14/20 11/23	14/20 12/22	28.8 ± 6.3 27.2 ± 4.6	55 (48–60) 50 (43–65)	T7
Ibrahim et al. [21] 2020	Jeddah, Saudi Arabia	Double-blinded, Randomized Controlled trial	ESPB OSTAPB	21 21	36.67 ± 9.4 37.5 ± 7.54	6/15 7/14	12/9 10/11	29.52 ± 4.0 28.91 ± 6.3	63.42 ± 8.64 65.33 ± 9.34	T8
Sahu et al. [22] 2021	Odisha,India	Single Blind Randomized Clinical trial	ESPB OSTAPB	30 30	41.3 ± 11.8 40.3 ± 11.1	19/11 16/14	N/A N/A	23.2 ± 3.3 23.4 ± 2.4	55.8 ± 10.3 56.0 ± 10.1	T7
Ozdemir et al. [23] 2021	Konya, Turkey	Randomized, Single-blind Study	ESPB OSTAPB	32 32	45. ± 10.5 45 ± 11.4	16/16 20/12	15/11 17/7	27.3 ± 2.9 26.8 ± 2	42.6 ± 4.4 41.3 ± 3	T7
Mounika et al. [24] 2023	Bhubaneswar, India	Prospective Randomized Trial	ESPB OSTAPB	69 69	40 (31–55) 50(40–57)	33/36 25/44	48/21 41/28	N/A N/A	120 (90–120) 100 (90–120)	T7

### Quality assessment

**Fig. 2:**
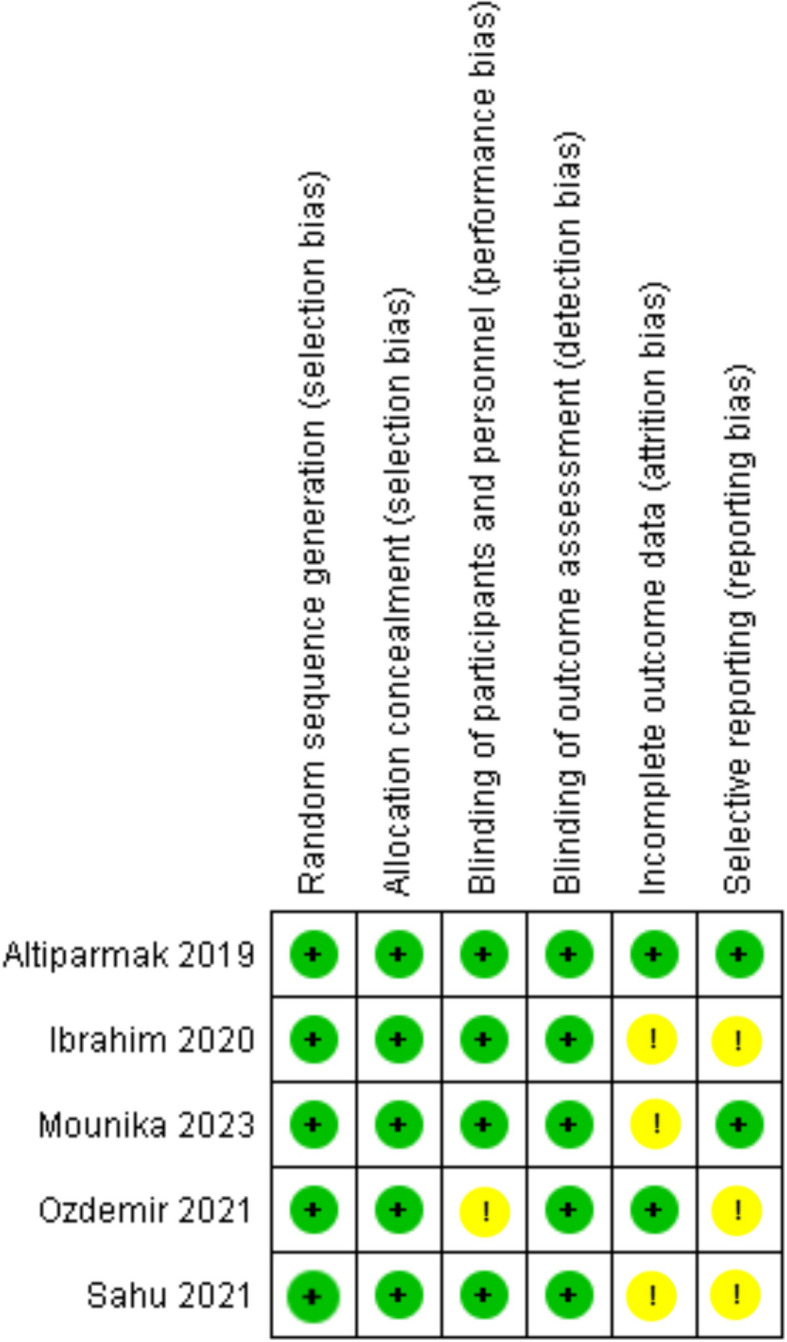
Risk of bias summary. The Cochrane “risk of bias” tool was used for quality assessment. Green for “no risk” and yellow for “unclear risk”

We assessed the quality of the 5 RCTs using the Cochrane risk of bias tool and overall, all the studies were found to have low risk of bias and are comprehensively shown in Fig. 2. This study by Altiparmak et al. [20] was deemed as high quality as it had low risk of bias in all the assessing criteria while, the remaining studies had only one or two unclear risks in performance, reporting or attrition bias. Publication Bias was not assessed as our study included only 5 studies because it is difficult to perform for less than 10 included studies which is the standardized criteria as the assessment is not reliable [25].

### Postoperative pain scores at 12 h and 24 h

**Fig. 3:**
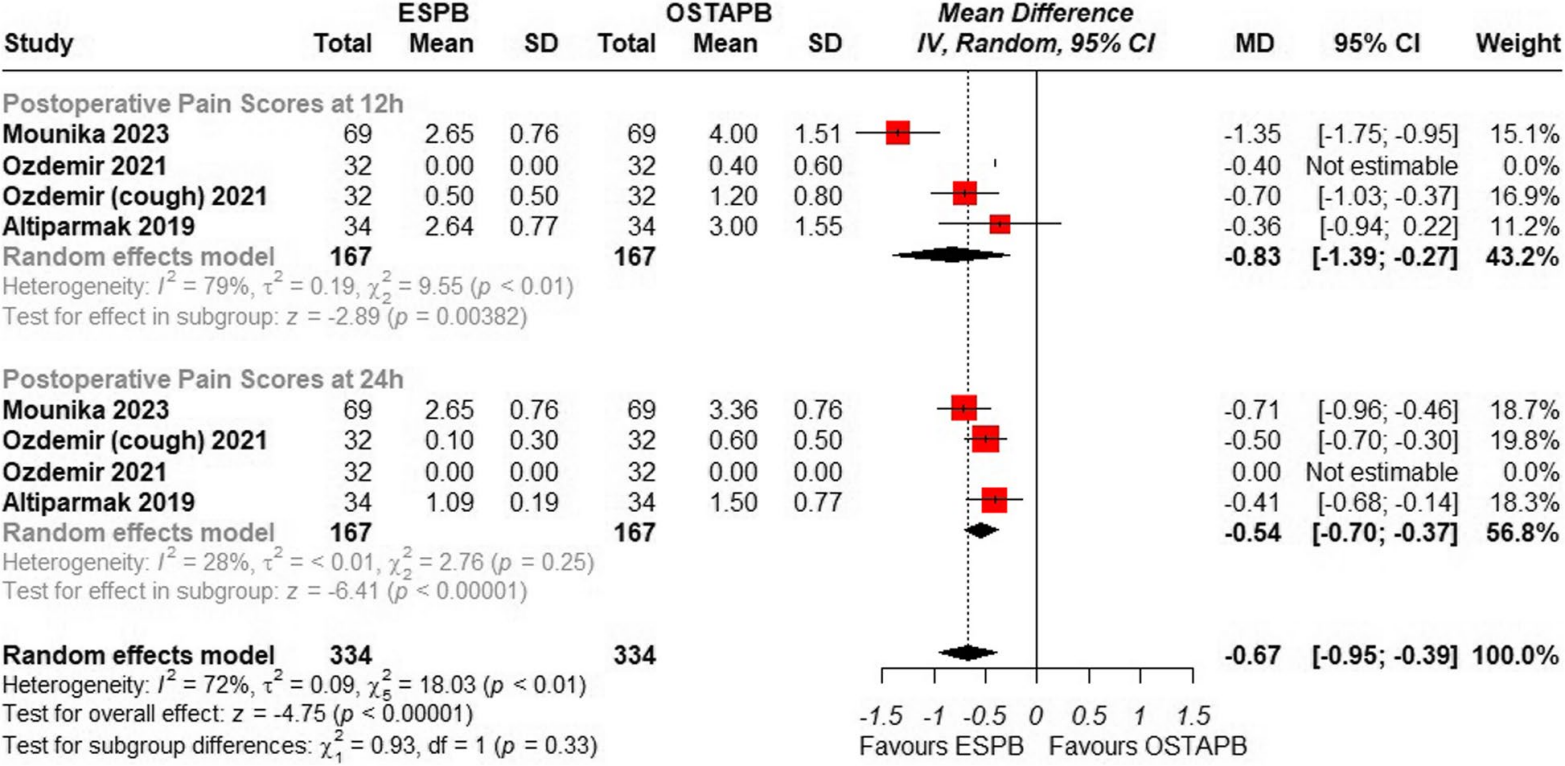
Forest plot of comparison: 1 ESPB vs OSTAPB, outcome: 1.1 Postoperative Pain Scores at 12 and 24 h. Subgroup analysis was performed with two subgroups of postoperative pain at 12 h and 24 h

**Fig. 4:**
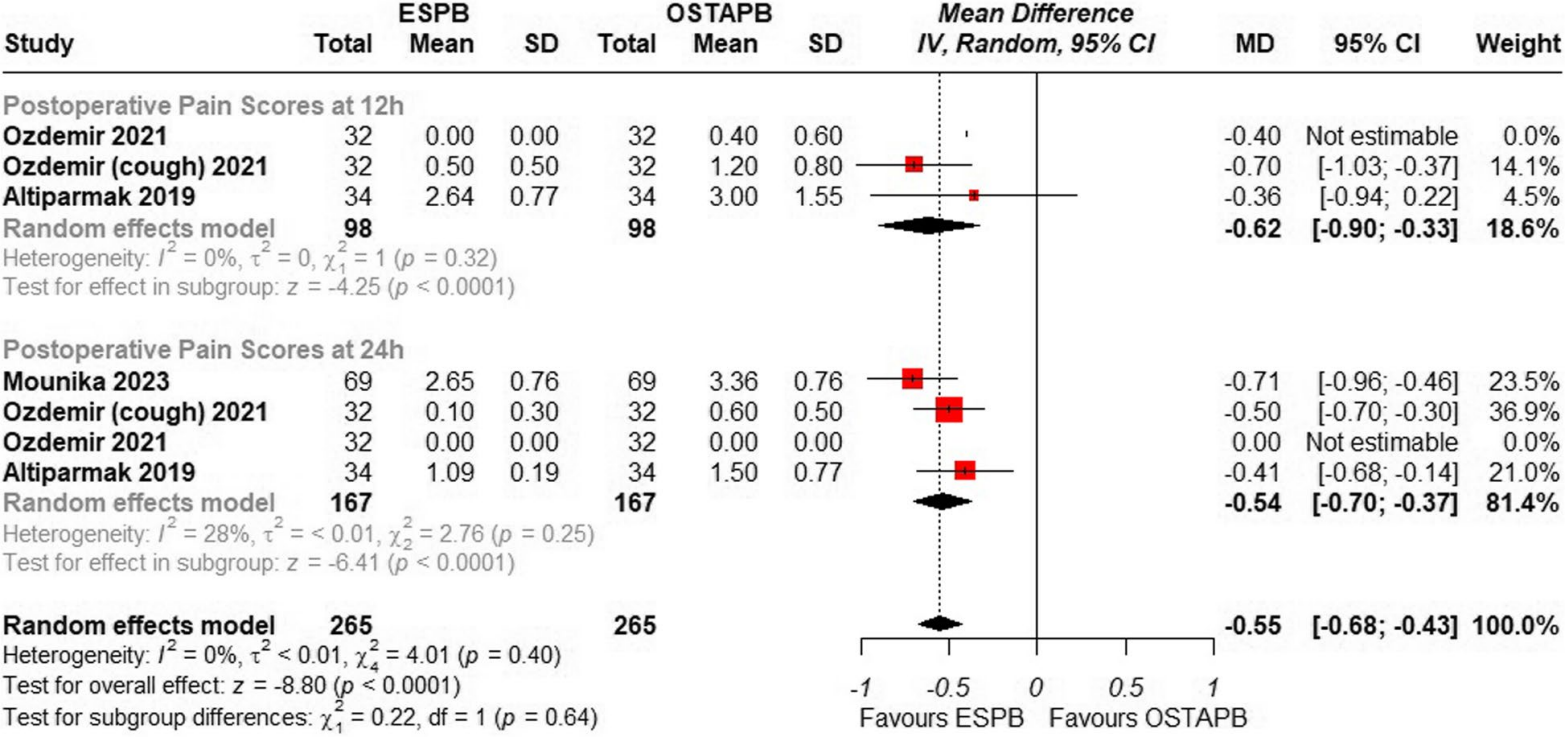
Forest plot of comparison: 1 ESPB vs OSTAPB, outcome: 1.2 Postoperative Pain Scores at 12 and 24 h (excluding Mounika et al.) Sensitivity analysis was performed by step wise removal of each study

Three studies [20, 22, 23] including a total of 270 patients investigated postoperative pain scores at 12 and 24 h using a numerical rating scale or visual analog scale. The study by Ozdemir et al. [22] the pain scores at both rest and coughing state while the study by Ibrahim et al. [21] was not included as it did not report Standard deviation. The pooled result demonstrated that the overall mean difference indicates a significant reduction in pain scores with ESPB compared to OSTAPB {MD = − 0.67; 95% CI: (− 0.95 to − 0.39); *p* < 0.001, I^2^ = 72%}, Fig. 3. Leave-one-out analysis was performed for the moderate heterogeneity, removing the study by Mounika et al. [23] for pain scores at 12 h the overall heterogeneity resolved to 0% and the overall result still reaches statistical significance {MD = − 0.55; 95% CI: (− 0.68 to − 0.43); *p* < 0.001, I^2^ = 0%}, Fig. 4. A detailed leave-one-out analysis can be seen in the Supplementary Fig. 1. Furthermore, a detailed subgroup analysis of the pain scores at multiple time intervals from 0 to 24 h was performed which found ESPB to be significantly better than OSTAPB {MD = − 0.98; 95% CI: (− 1.26 to − 0.69); *p* < 0.001, I^2^ = 85%} shown in Supplementary Fig. 2.

No significant subgroup differences were revealed between the ESPB and OSTAPB techniques for postoperative pain at 12- and 24-h intervals.

### Postoperative opioid consumption at 24 h

**Fig. 5:**
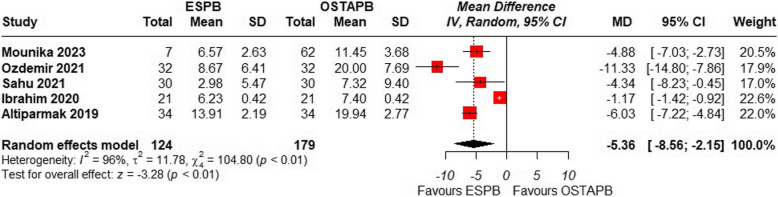
Forest plot of comparison: 1 ESPB vs OSTAPB, outcome: 1.3 Postoperative Opioid Consumption at 24 h. Opioid consumption at postoperative 24 h was significantly lower in the ESPB group than in the OSTAPB group

All five RCTs reported this outcome. To facilitate the process of data analysis, the doses of tramadol and fentanyl reported in the included studies were transformed into morphine-equivalent doses. This conversion was conducted based on prior scientific investigations that suggested 100 mg intravenous tramadol or 100 μg intravenous fentanyl to be comparable to 10 mg of intravenous morphine [26]. Pooled analysis of postoperative opioid consumption at 24 h showed a significant reduction in opioid consumption in the ESPB group compared to OSTAPB with a large effect size {MD = − 5.36; 95% CI: (− 8.56 to –2.15); *p* < 0.001, I^2^ = 96%}, Fig. 5.

**Fig. 6:**
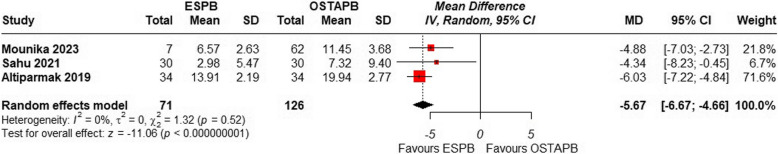
Forest plot of comparison: 1 ESPB vs OSTAPB, outcome: 1.4 Postoperative Opioid Consumption at 24 h (excluding Ibrahim et al. and Ozdemir et al.). Sensitivity analysis was performed by step wise removal of each study

For the high heterogeneity leave-one-out analysis was performed which revealed these studies by Ibrahim et al. [21] and Ozdemir et al. [22] to be the outliers. After removing, the heterogeneity resolved to 0% and the overall pooled result still showed a significant reduction of opioid administration with ESPB {MD = − 5.67; 95% CI: (− 6.67 to –4.66); *p* < 0.001, I^2^ = 0%}, Fig. 6. A detailed leave one out analysis has been shown in Supplementary Fig. 3.

### Intraoperative opioid consumption

**Fig. 7:**
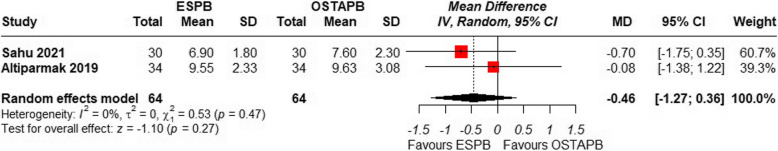
Forest plot of comparison: 1 ESPB vs OSTAPB, outcome: 1.5 Intraoperative Opioid Consumption

Two studies reported this outcome [20, 24]. Intraoperative opioid consumption showed a small statistically non-significant effect size in favor of ESPB group {MD = − 0.46; 95% CI: (− 1.27 to –0.36); *p* = 0.27, I^2^ = 0%}, Fig. 7. However, the limited number of studies may have affected the precision of the estimate for this outcome.

### Postoperative nausea and vomiting

Four studies were included in the analysis of this outcome. While Ibrahim et al. [21] reported nausea and vomiting at PACU and 24-h intervals separately, the combined result was not statistically significant.

**Fig. 8:**
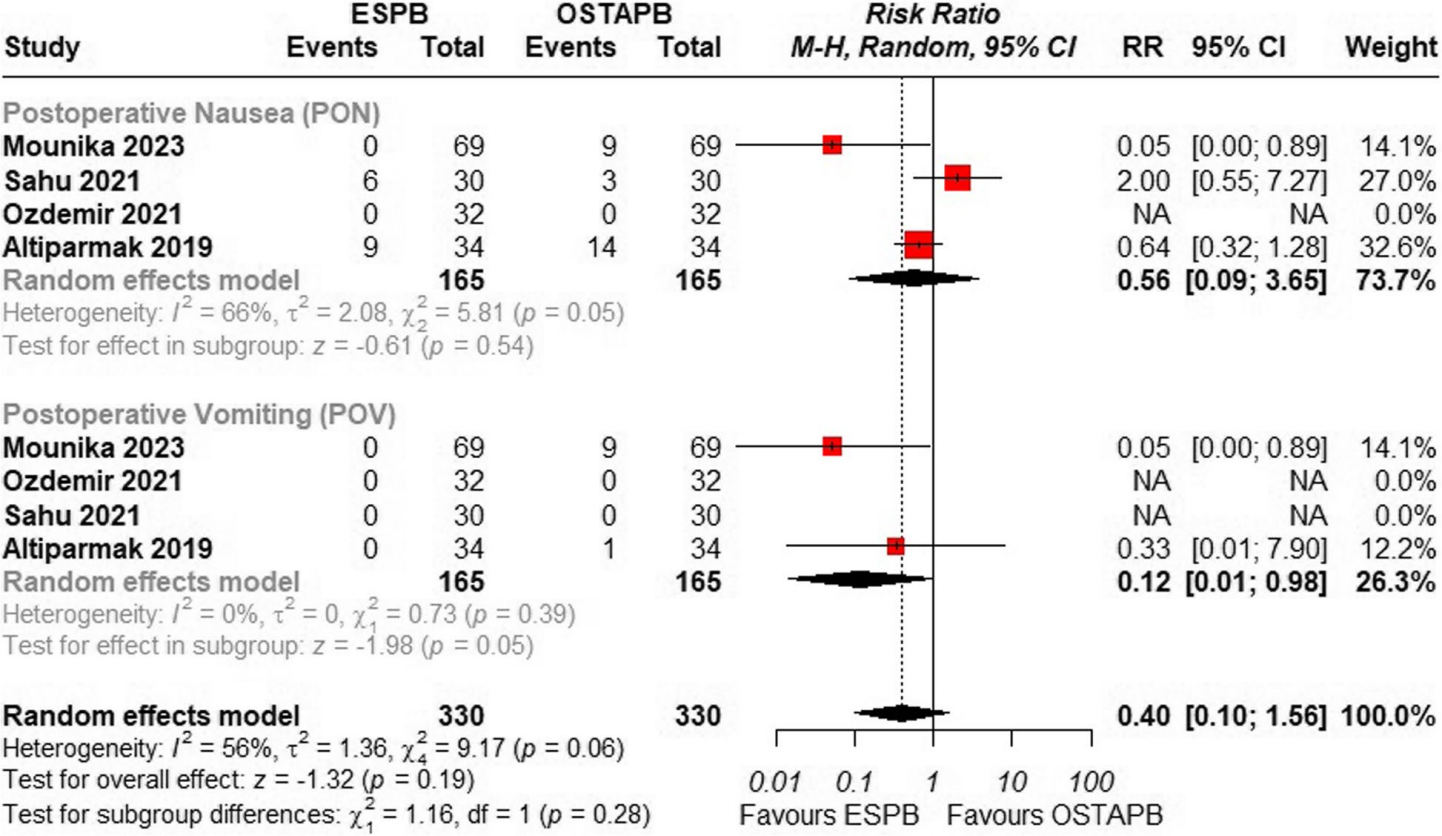
Forest plot of comparison: 1 ESPB vs OSTAPB, outcome: 1.6 Postoperative Nausea Vomiting. Subgroup analysis was performed based on two subgroups of postoperative nausea and postoperative vomiting. Incidence of post operative vomiting was significantly less in ESPB group

The overall risk ratio showed no significant difference between the two blocks {RR = 0.40, 95% CI (0.10 to 1.56), *p* = 0.19; I^2^ = 56%}, Fig. 8.

Subgroup analysis was performed based on the type of outcome reported across studies into subgroups of postoperative nausea (PON), postoperative vomiting (POV) and combined postoperative nausea vomiting (PONV). No significant subgroup differences were revealed between the ESPB and control for post operative nausea and vomiting (*p* = 0.28), Fig. 8.

## Discussion

ESPB is a regional anesthesia technique that involves the injection of local anesthetic agents into the erector spinae plane, a plane of tissue located between the transverse process of the vertebrae and the erector spinae muscle. The mechanism of action of ESPB is still under investigation; however, it has been shown to be an effective strategy for pain control in specific clinical settings. It has been recently evident as a promising alternative for the management of thoracic neuropathic pain [11, 27]. On the other hand, OSTAPB involves the administration of local anesthetics to the nerves that supply the anterior abdominal wall. The technique is performed by injecting local anesthetics into the plane between the internal oblique and transversus abdominis muscles. OSTAPB has been shown to be effective in reducing postoperative pain in several types of surgeries, including laparoscopic cholecystectomy and abdominal hysterectomy [28, 29].

The combined analysis of acute postoperative pain scores divided into two subgroups at 12 and 24 h showed ESPB to be superior of the two blocks as the pooled results were statistically significant in contrary to a previous meta-analysis by Koo et al. [30] which found the two techniques to be comparable for analgesia but the analysis consisted on a limited number of studies. For the associated moderate heterogeneity, leave-one-out analysis removing the study by Mounika et al. [23] resolved the heterogeneity concern. The presence of intense pain following surgery poses a considerable risk for the emergence of long-term chronic pain as a prior study conducted with patients undergoing laparoscopic cholecystectomy (LC) [31] revealed a connection between early visceral pain and the development of chronic pain. In this regard ESPB represents a promising option for perioperative analgesia and may play an important role in reducing chronic post-surgical pain after LC. While the results were statistically significant, whether this difference meets the minimal clinically important difference (MCID) for postoperative pain relief on pain scale was unassessable. However, it's crucial to assess whether the reduction in risk translates to meaningful improvements in patient recovery and satisfaction.

Moreover, OSTAPB only produces sensory blocks in the somatic branches of the spinal nerves. Thus, ESPB may have a potential analgesic mechanism for visceral pain and is expected to provide better analgesia than OSTAPB [32]. 

Additionally, an extended version of this outcome was analyzed based on subgrouping by taking into account all the different time intervals reported across included studies from 0 up to 24 h showing significantly better pain reduction with ESPB. This helped us get a much deeper understanding as it was not performed earlier and since pain is a very subjective experience that can vary over time, analyzing pain scores at different intervals allows for a more nuanced understanding of its trajectory, and increases the statistical power of this study. Of note, only three studies reported pain scores at different time frames which highlights the need for further in-depth studies.

As for the secondary outcomes, postoperative opioid consumption at 24 h was significantly reduced with the intervention of ESPB compared to OSTAPB consistent with the finding of the previous meta-analysis [13]. Moreover, for the concern of high heterogeneity, we performed sensitivity analysis removing two outlier studies which reduced the heterogeneity to 0%. There are various considerations for such high heterogeneity found in our study that includes variability in Block Techniques – Differences in how ESPB and OSTAPB are performed, including variations in the level of injection, volume, and concentration of local anesthetics, may contribute to heterogeneity. There could be differences in patient populations such as variation in patient characteristics like age, BMI, comorbidities, and pain thresholds across studies can influence postoperative pain scores and analgesic consumption. Variability in Surgical Techniques for differences in laparoscopic cholecystectomy methods (e.g., standard vs. single-incision surgery, use of electrocautery vs. harmonic scalpel) could affect pain levels and influence the effectiveness of regional blocks. Diverse outcome measurement methods like pain assessment scales, time points for evaluation, and criteria for rescue analgesia use may not be standardized across studies, leading to inconsistencies in reported outcomes. Heterogeneity in Control and Additional Analgesia is another set of variations in multimodal analgesic regimens, including the use of opioids, NSAIDs, or adjuncts like dexmedetomidine, may contribute to differences in reported pain relief and overall effect sizes. Since we only used RCTs in our study, differences in study methodologies (RCTs vs. observational studies), sample sizes, and blinding methods, did not contributed to the risk of bias that could have lead to variability in results. However, publication and reporting bias due selective reporting of positive results in some studies may lead to an overestimation or underestimation of the true effects, adding to heterogeneity which is undesirable but non-modifiable risk factor.

The importance of effective pain management cannot be overstated, particularly in the context of the opioid epidemic. ESPB seems to be particularly useful in reducing opioid consumption, which is associated with an increased risk of adverse events such as respiratory depression, sedation, and nausea as well as prolonged length of hospital stay, and higher 30-day readmission rates [33, 34]. In the realm of multimodal pain relief techniques, the findings of this analysis, highlighting the effectiveness of ESPB in reducing the need for opioids, could potentially contribute to achieving pain relief while minimizing opioid usage.

Unexpectedly for Intraoperative opioid consumption no significant difference was seen between the two groups as the pooled result was statistically insignificant with a small effect size. Individuals can experience varying levels of pain and have different needs for opioids, which can be influenced by factors like genetics, previous exposure to opioids, and psychological aspects [35]. The natural differences among patients along with limited studies analyzing this outcome might have concealed any potential disparities in opioid usage between the ESPB group and the OSTAPB group.

The postoperative nausea and vomiting effect size difference was not significantly different between the two techniques. Postoperative nausea and vomiting (PONV) are common adverse events occurring after surgery, with an estimated incidence of 30%. However, in high-risk patients, the incidence can be as high as 80% [36]. According to the fourth consensus guideline for PONV management, opioids have been recognized as a risk factor for PONV in adults, and their association with PONV demonstrates a dose-dependent relationship [37]. Hence, more new studies evaluating this outcome need to be published as ESPB may be found to be a safer choice in future due to the reduction in opioid usage more than OSTAPB.

In conclusion, both ESPB and OSTAPB are effective techniques for reducing postoperative pain. While there is more and more evidence to suggest that ESPB may be more effective than OSTAPB, more research is needed to confirm this. Ultimately, the choice of technique will depend on a range of factors, including the type of surgery, the patient's medical history, and the clinician's experience and preference.

## Limitations

In the end, there were some limitations with this study that cannot be avoided and should be considered when referring to the outcomes of this meta-analysis. Firstly, moderate to high heterogeneity was observed in some outcomes, however, to address this, we conducted sensitivity and subgroup analysis. This could be attributed to variations in ESPB approaches among the included studies, such as differences in block techniques (timing, positions), levels of vertebra approached, types of local anesthetics, their concentrations, and volumes. The limited number of studies and the small sample size in certain comparisons may affect the generalizability of the findings. The specific patient population may also limit the generalizability of the findings as among the five studies included, only one study (Altiparmak et al.) [20] included individuals over the age of 65, which means the findings can hardly be generalized to this age group. Additionally, there were variations in types of analgesia used and although we did convert the different doses of the various analgesics into morphine equivalent doses it could have an impact on the overall results. Lastly, more and more new studies need to be conducted to ascertain the optimal dose and type of anesthetic and to overall establish Erector spinae plane block (ESPB) as the preferred technique in performing regional nerve blocks specifically for laparoscopic cholecystectomy. Also, we were unable to analyze publication bias as the number of studies were less than 10 in the final analysis.

## Conclusion

In conclusion, overall erector spinae plane block (ESPB) seems to be the treatment of choice in the context of the acute postoperative pain and opioid usage in laparoscopic cholecystectomy compared to oblique subcostal transversus abdominis plane block (OSTAPB). Moreover, our analysis also deems it to be a safer option especially for postoperative vomiting adverse events. Besides, further studies are needed comparing the above two blocks to confirm our findings and establishing the added pain relief associated with ESPB in laparoscopic cholecystectomy which will help in mitigating the usage of opioids for analgesia.

Further research more prominently should include more large-scale, multi-center RCTs to substantiate the superiority of ESPB over OSTAPB in various surgical procedures and we recommend for reducing variability in future studies.

## Supplementary Information

Supplementary Material 1. Supplementary Figure. 1: Step wise leave-one-out analysis for Post operative Pain Scores at 12 and 24 hours

Supplementary Material 2. Supplementary Figure. 2. Forest plot of comparison: 1 ESPB vs OSTAPB, outcome: 1.2 Postoperative Pain Scores. Subgroup analysis was performed. Subgroups were made based on different time intervals from 0 up to 24 hours postoperatively

Supplementary Material 3. Supplementary Figure. 3: Step wise leave-one-out analysis for postoperative opioid consumption at 24 hours

Supplementary Material 4. Supplementary Table S1: Detailed Search Strategy. Supplementary Table S2: List and Definition of Outcomes

## Data Availability

The dataset supporting the conclusions of this article is included within this article.

## Abbreviations

CI: Confidence Interval
ESPB: Erector spinae plane block
LA: Local Anesthesia
LC: Laparoscopic cholecystectomy
MD: Mean difference
OSTAPB: Oblique subcostal transversus abdominis plane block
PONV: Postoperative nausea and vomiting
PRISMA: Preferred Reporting Items for Systematic Reviews and Meta Analyses
RCTs: Randomized controlled trials
RR: Risk Ratio
