# Supplementary figures and images for: Redefining pain management: investigating the efficacy and safety of erector spinae plane block and oblique subcostal transversus abdominis plane block in laparoscopic cholecystectomy – a meta analysis of randomized controlled trials
Source: BMC Anesthesiol. 2025 Apr 16;25:182. doi: 10.1186/s12871-025-03059-1 (PMC12001665; doi:10.1186/s12871-025-03059-1)

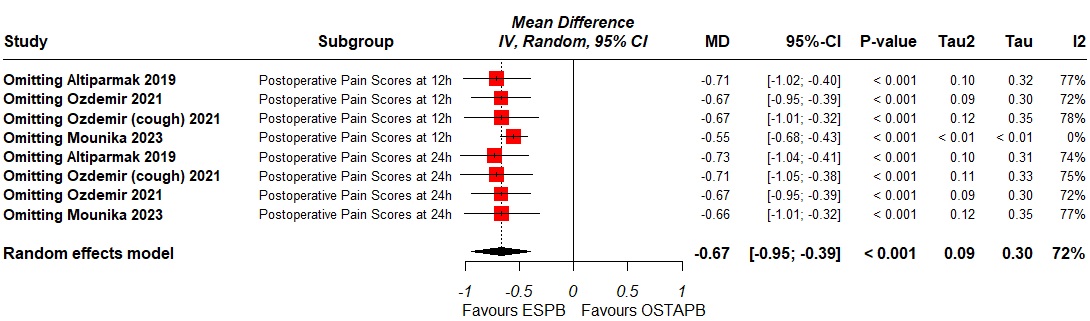

Supplement: Supplementary file 1 — Supplementary Material 1. Supplementary Figure. 1: Step wise leave-one-out analysis for Post operative Pain Scores at 12 and 24 hours [file 12871_2025_3059_MOESM1_ESM.jpg]

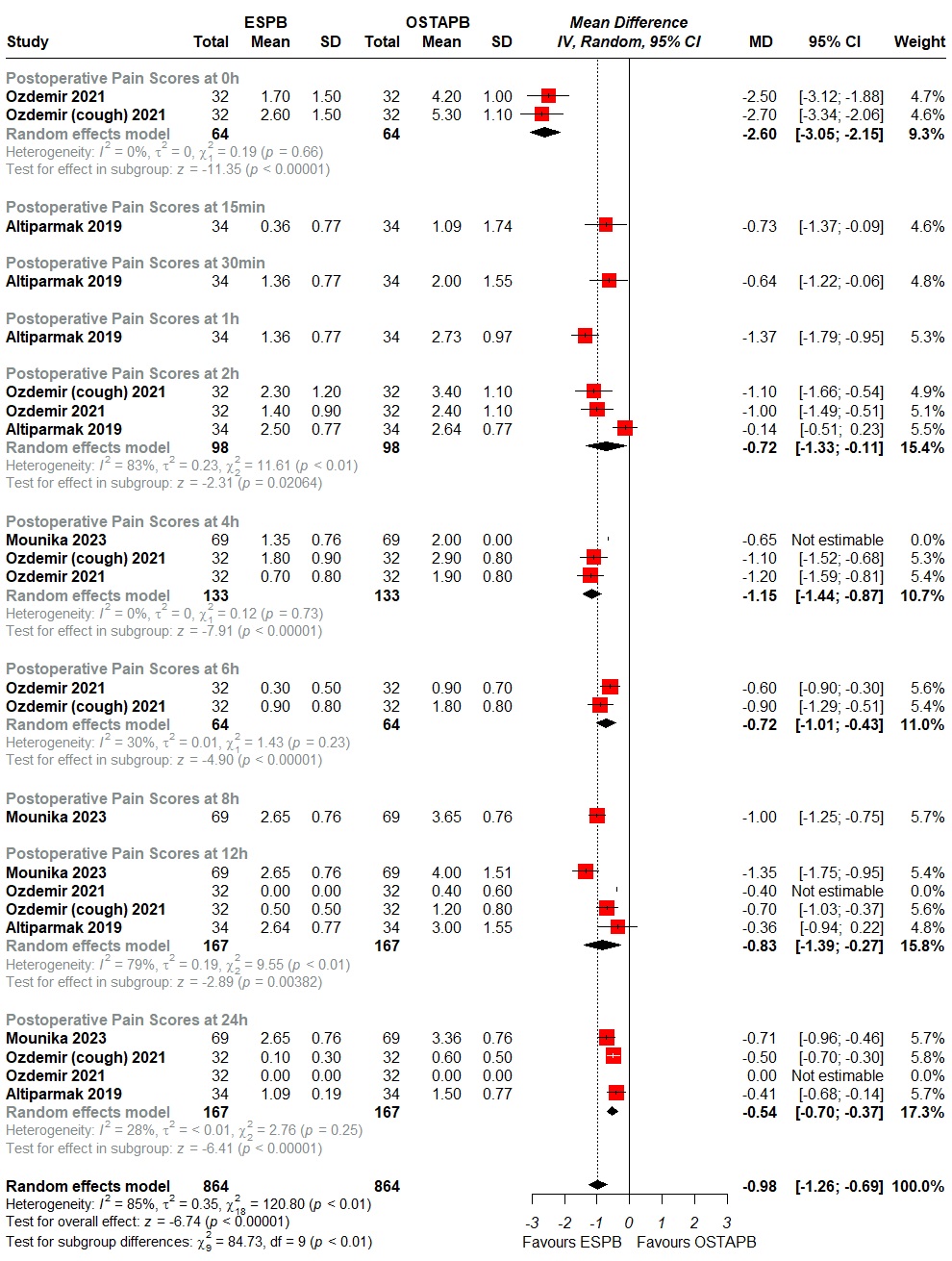

Supplement: Supplementary file 2 — Supplementary Material 2. Supplementary Figure. 2. Forest plot of comparison: 1 ESPB vs OSTAPB, outcome: 1.2 Postoperative Pain Scores. Subgroup analysis was performed. Subgroups were made based on different time intervals from 0 up to 24 hours postoperatively [file 12871_2025_3059_MOESM2_ESM.jpg]

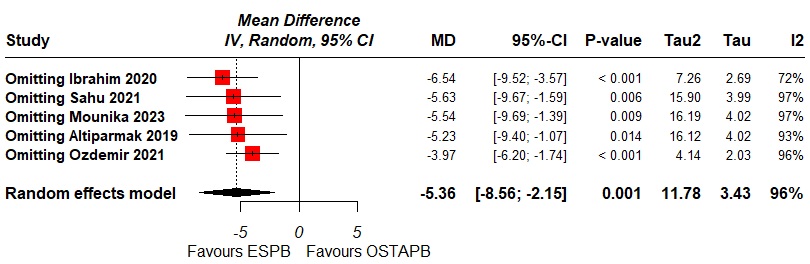

Supplement: Supplementary file 3 — Supplementary Material 3. Supplementary Figure. 3: Step wise leave-one-out analysis for postoperative opioid consumption at 24 hours [file 12871_2025_3059_MOESM3_ESM.jpg]
