# Supplementary material for: Redefining pain management: investigating the efficacy and safety of erector spinae plane block and oblique subcostal transversus abdominis plane block in laparoscopic cholecystectomy – a meta analysis of randomized controlled trials
Source: BMC Anesthesiol. 2025 Apr 16;25:182. doi: 10.1186/s12871-025-03059-1 (PMC12001665; doi:10.1186/s12871-025-03059-1)
Supplement: Supplementary file 4 — Supplementary Material 4. Supplementary Table S1: Detailed Search Strategy. Supplementary Table S2: List and Definition of Outcomes [file 12871_2025_3059_MOESM4_ESM.docx]

Supplementary Table S1. Detailed search strategy

| **Database** | **Detailed Search String** |
| --- | --- |
| **Pubmed** | ("erector"[All Fields] OR "erectores"[All Fields] OR "erectors"[All Fields]) AND "Spinae"[All Fields] AND ("block"[All Fields] OR "blocked"[All Fields] OR "blocking"[All Fields] OR "blockings"[All Fields] OR "blocks"[All Fields]) AND (("oblique"[All Fields] OR "obliquely"[All Fields] OR "obliqueness"[All Fields] OR "obliques"[All Fields] OR "obliquities"[All Fields] OR "obliquity"[All Fields]) AND ("subcostal"[All Fields] OR "subcostally"[All Fields]) AND ("abdominal muscles"[MeSH Terms] OR ("abdominal"[All Fields] AND "muscles"[All Fields]) OR "abdominal muscles"[All Fields] OR ("transversus"[All Fields] AND "abdominis"[All Fields]) OR "transversus abdominis"[All Fields]) AND ("block"[All Fields] OR "blocked"[All Fields] OR "blocking"[All Fields] OR "blockings"[All Fields] OR "blocks"[All Fields])) AND ("cholecystectomy, laparoscopic"[MeSH Terms] OR ("cholecystectomy"[All Fields] AND "laparoscopic"[All Fields]) OR "laparoscopic cholecystectomy"[All Fields] OR ("laparoscopic"[All Fields] AND "cholecystectomy"[All Fields])) |
| **Cochrane** | “Erector Spinae Plane Block” OR "ESPB“ AND Oblique subcostal transversus abdominis plane block” OR "OSTAPB" AND “Laparoscopic Cholecystectomy” |

**Supplementary Table S2: List and Definition of Outcomes**

| **Outcomes** | **Definition** |
| --- | --- |
| 1. Post operative pain scores | **Numerical Rating Scale (NRS):** Participants rate their experience on a scale from 0 to 10, where 0 is no sensation and 10 is the highest intensity.  **Visual Analog Scale (VAS):** Participants mark a point on a line (usually 10 cm long) to indicate their experience's intensity, with one end representing absence and the other end representing maximum intensity.  an NRS score of 6 to 7 is moderate pain, and an NRS score of 8 to 10 indicates severe pain while, VAS uses a scale from 0 (no pain) to 10 (worst pain imaginable)[1,2]. |
| 2. Postoperative opioid consumption at 24 hours | Total amount of opioid pain medication a patient has taken within the first 24 hours after surgery |
| 3. Intraoperative opioid consumption | Amount of opioid pain medication administered to a patient during the course of a surgical procedure. |
| 4. Postoperative nausea (PON) and vomiting (POV) | Postoperative nausea and vomiting (PONV) is defined as any nausea, retching, or vomiting occurring during the first 24–48 h after surgery [3]. |

References

1. Cho S, Kim YJ, Lee M, Woo JH, Lee HJ. Cut-off points between pain intensities of the postoperative pain using receiver operating characteristic (ROC) curves. BMC Anesthesiology. 2021 Jan 25;21(1).

2. Breivik H, Borchgrevink PC, Allen SM, et al. Assessment of pain. British Journal of Anaesthesia. 2008;101:17–24. doi:10.1093/bja/aen103

3. Pierre S, MD, Whelan R. Continuing Education in Anaesthesia Critical Care & Pain, Volume 13, Issue 1, February 2013, Pages 28–32, https://doi.org/10.1093/bjaceaccp/mks046
